# Childhood adversity and sex trafficking experiences linked to economic and psychological outcomes for U.S. survivors of commercial sexual exploitation

**DOI:** 10.3389/fpsyt.2026.1774509

**Published:** 2026-03-13

**Authors:** Courtney Furlong, James Benjamin Hinnant

**Affiliations:** 1 Psychology Department, Huntingdon College, Montgomery, AL, United States; 2 Department of Human Development and Family Science, Auburn University, Auburn, AL, United States

**Keywords:** adverse childhood experiences, commercial sexual exploitation, educational achievement, sex trafficking, socioeconomic disadvantage, survivor outcomes

## Abstract

**Introduction:**

This investigation sought to develop a deeper understanding of the lived experiences of survivors of commercial sexual exploitation by considering the links between childhood background, trafficking experiences, and outcomes.

**Methods:**

To that end, 350 survivors of commercial sexual exploitation living in the US were surveyed. Guided by ecological and life course frameworks, variables included adverse childhood experiences, socioeconomic disadvantage, educational achievement, trafficking experiences, and several outcomes, including income, employment status, posttraumatic stress symptoms, dignity, and sobriety.

**Results:**

Results suggested that adverse childhood experiences were strongly linked to more severe exploitation and poorer outcomes. Educational achievement improved income and employment without reducing trafficking severity. Socioeconomic disadvantage showed no significant risk, contradicting prior research. Further, more severe trafficking experiences were significantly linked to poorer economic stability. Findings went on to suggest that trafficking-related experiences may play a mediating role in the negative effects of early adversity on income, employment, and sobriety.

**Discussion:**

This investigation highlights the diverse and individualized needs of survivors of commercial sexual exploitation, indicating that tailored interventions are essential for addressing the complex interplay of childhood adversity, trafficking experiences, and recovery trajectories.

## 1 Introduction

When anything of value (i.e., money, food, shelter, drugs, clothing, etc.) is exchanged for a sex act (i.e., prostitution, exotic dancing, pornography, illicit massage, webcamming, etc.), it is termed commercial sexual exploitation. Sex trafficking involves the commercial sexual exploitation of a person through force, fraud, or coercion (1). An individual under the age of 18 who experiences commercial sexual exploitation is a victim of sex trafficking without needing to demonstrate force, fraud, or coercion. Individuals who experience commercial sexual exploitation report physical and sexual assaults, sexually transmitted infections, and other medical conditions related to high-risk sexual contact, as well as mental health disorders and even murder (2–4). Factors such as poverty, family violence, neglect, academic failure, and a history of childhood sexual abuse create vulnerabilities to commercial sexual exploitation (2, 5–8). To better understand the lived experiences of survivors of commercial sexual exploitation and to identify indicators that may assist survivors as they exit exploitation, this investigation will consider the relationships between the childhood backgrounds of survivors, their trafficking experiences, and their long-term outcomes.

## 2 Literature review

### 2.1 The terminology of commercial sexual exploitation

“Sex trafficking,” “prostitution,” “sex work,” “domestic minor sex trafficking,” and “modern day slavery” are just a few of the terms associated with commercial sexual exploitation. Sex trafficking is a form of commercial sexual exploitation that involves the use of force, fraud, or coercion (22 U.S.C. § 7102). In a study representing 1,264 unique survivors of commercial sexual exploitation, 75% reported that they had experienced exploitation under a third party at some point (i.e., were victims of sex trafficking; 6). Based on feedback from focus group members, we will use the broader term, “commercial sexual exploitation,” throughout the investigation (9). To promote strengths-based language, the term “survivor” (rather than victim) will be used to describe study participants. Because we wish to acknowledge the reality of victimization and yet do not desire to diminish the agency of survivors, the terms “entry” and “exit” are intended to be neutral terms related to exploitation experiences.

### 2.2 Multisystem frameworks: ecological and life course theories

Sex trafficking research remains a relatively new field, and as such, testable theoretical models of commercial sexual exploitation are lacking (10, 11). Even so, ecological theory (12) and life course theory (13) suggest promising pathways for understanding entry into and exit out of exploitation. Both theories complement each other by providing a comprehensive understanding of human development through the interplay of individual life trajectories and environmental contexts.

Ecological theory offers a framework that explains how individual, relational (familial or microsystem), social (community or exosystem), and societal (cultural experiences or macrosystem) factors shape an individual. Vulnerability and risk to commercial sexual exploitation—as well as solutions and interventions—are embedded in all levels of the system (6, 14). For example, relevant risk factors at the individual level might include educational achievement and mental health. Adverse childhood experiences and childhood socioeconomic disadvantage, as well as educational achievement, may exist at both the relational and social levels.

Time, culture, context, and the interdependence of familial relationships are additional considerations provided by life course theory (13). Life course theory acknowledges that agency only exists within the context of relationships, events, and historical time and place. Further, these contexts evolve throughout a person’s life, often via transitions and turning points. Life course theory has been mentioned in several studies of commercial sexual exploitation (as in 6, 15–18). The complex and compounding relationships between childhood background, trafficking experiences, and outcomes provide challenges as well as opportunities for survivors of commercial sexual exploitation to realize their promising futures.

### 2.3 Childhood background

#### 2.3.1 Adverse childhood experiences

Felitti et al. (19) conducted the Adverse Childhood Experiences (ACES) Study, which considered the relationship between negative childhood experiences, like abuse and divorce, and long-term health outcomes. Since then, ACES have been linked to heart disease, diabetes, and cancer (19, 20). Children with four or more ACES are more likely to experience depression, sexually transmitted diseases, substance abuse, alcoholism, obesity, and suicide attempts (21).

Approximately two-thirds of survivors of commercial sexual exploitation report experiencing four or more ACES (22, 23). The most common ACES for survivors of commercial sexual exploitation include sexual abuse, physical abuse, and emotional abuse (22–24). The rate of childhood sexual abuse for survivors of commercial sexual exploitation ranges from 60-90% (5, 6, 24–26). Childhood sexual abuse is significantly linked to commercial sexual exploitation, and 70% of survivors believe that childhood sexual abuse influenced their entry into commercial sexual exploitation (26–29). The rate of childhood physical abuse for survivors of commercial sexual exploitation ranges from 53-80% (5, 24, 25, 28). While the link between ACES and commercial sexual exploitation is well documented, the association between the severity of exploitation as well as long-term outcomes has not been considered.

#### 2.3.2 Educational achievement

Educational achievement is necessary to secure legitimate, skilled employment. According to the United States Census Bureau (30), 8.9% of the general American public has less than a high school diploma or equivalent. However, individuals who have experienced commercial sexual exploitation are disproportionately represented among those with lower educational achievement compared to the general population, establishing vulnerability for abuse and exploitation (6, 26, 31–33). In a sample of adult female survivors of commercial sexual exploitation (*n* = 1,264), We found that 38.1% had less than a high school diploma or equivalent (6). The findings suggested that educational achievement may serve as an important protective factor and decrease vulnerability to, and severity of, exploitation as it was linked to older ages of entry into commercial sexual exploitation, shorter lengths of commercial sexual exploitation, lower numbers of children, and lower arrest rates (6).

#### 2.3.3 Childhood socioeconomic disadvantage

Generally, one in seven children in the United States is living in poverty (34). In 2024, approximately 24% of children in the United States received public assistance, and 12% of people in the US received Supplemental Nutrition Assistance Program benefits (SNAP, formerly food stamps; 35, 36). Poverty is frequently touted as the primary vulnerability for commercial sexual exploitation (37, 38). As early as 1910, researchers were suggesting that commercial sexual exploitation (i.e., prostitution) was primarily caused by poverty and food insecurity (39). Women and children are disproportionately impacted by poverty, and women are less likely to be able to access the limited number of jobs available (34, 40–42), but the direct link between socioeconomic disadvantage and commercial sexual exploitation is unclear.

### 2.4 Commercial sexual exploitation experiences

#### 2.4.1 Age of entry into commercial sexual exploitation

Age of entry into commercial sexual exploitation spans from less than one year up to 55 years old, though the average ages range from 15 to 22 (5, 6, 33, 43, 44). The most salient window of vulnerability exists between the ages of 18 and 20 (5, 6, 33, 44). In our study of 1,264 adult survivors, we found a link between early ages of entry into commercial sexual exploitation, childhood sexual abuse, and low educational achievement (6). In turn, earlier ages of entry were linked to higher numbers of arrests and longer experiences of commercial sexual exploitation.

#### 2.4.2 Length of commercial sexual exploitation

Length of commercial sexual exploitation can range from less than one day to 46 years (5, 6, 43, 45). The Counter Trafficking Data Collaborative (45) dataset, the largest human trafficking open-source dataset representing 222,825 individual cases of global human trafficking, suggests that the average length of exploitation of any kind is two years. However, Kramer (43) found that the length of commercial sexual exploitation for American females averaged 8.7 years, while our 2024 investigation of adult female survivors found that the average length was 12 years (6). We went on to link earlier ages of entry, higher numbers of arrests, and higher numbers of children with longer experiences of exploitation (6).

#### 2.4.3 Trafficking Experiences

The stories of survivors of commercial sexual exploitation are featured in news segments, written about in books and magazine articles, and spotlighted in big-budget movies. While these stories represent interesting case studies, developing an understanding of the collective experiences of survivors of commercial sexual exploitation is necessary to prosecute crimes committed against them and establish programming to meet the unique needs of each. Indeed, there are several burgeoning domestic and international movements to breach this data gap, like efforts by the United States Department of Justice (DOJ), the United States Department of Health and Human Services (HHS), the United States Department of Homeland Security, and the International Organization on Migration (IOM; 46–49).

Polaris (50), a partner with IOM in the CTDC (45), reports that the top five forms of force, fraud, and coercion for survivors of sex trafficking in the United States include exploitation involving substance use, physical violence, sexual violence, intimidation with weapons, and emotional abuse, respectively. Individuals in prostitution in the United States in Farley et al.’s (2) study reported physical assault (82%), rape (73%), threats with weapons (78%), and being raped more than five times (59%). There appear to be many similarities across sexual exploitation experiences, but there are also significant differences, and it is unclear if, and how, these differences affect the outcomes of survivors of commercial sexual exploitation.

### 2.5 Outcomes

#### 2.5.1 Financial and employment stability

The term commercial sexual exploitation suggests financial manipulation and control as a paramount element of the victim experience. Whether controllers withhold earnings or enforce debt bondage, survivors of commercial sexual exploitation may find it difficult to establish financial independence, especially immediately following exit from exploitation (51). Dalla’s (52) qualitative analysis of 18 individuals living in the United States three years post support services acknowledged that the ability to acquire a living wage is of principal importance to avoiding cycling back into commercial sexual exploitation.

Survivors bear costs associated with victimization. One European study estimated the long-term economic burden of commercial sexual exploitation to be the equivalent of nearly $400,000.00 USD per victim (53). Costs may be related to medical and mental healthcare expenses, loss of work productivity, relocation costs, and criminal justice activities. Further, the inability to invest in one’s future may lead to extreme poverty as one ages (41).

A survivor of commercial sexual exploitation from Dalla’s (52) investigation, named Marlee, explains:

*Before [when I was experiencing commercial sexual exploitation], I just lived day by day, didn’t think about the future. And here it is I’m just starting to work—and keep in mind just about ready to retire. You can’t hustle all your life. What are you gonna do when you get too old, you know? You can’t draw Social Security. I don’t have really anything in Social Security and I’m 40 years old! So I’m really mad at myself about that.*

The “costs” associated with commercial sexual exploitation (e.g., medical and mental healthcare, criminal justice activities) may also make it challenging to acquire and maintain stable employment. Dalla’s (52) investigation determined that meaningful employment was one of the primary indicators for individuals to remain out of commercial sexual exploitation. Results suggested that employment provided financial support as well as improved self-confidence, developed job skills, and filled the time void following initial exit. Participants explained that acquiring jobs was not problematic, and though first employment opportunities were generally entry-level jobs, participants reported that they quickly moved into higher-paying positions. For those who cycled back into exploitation, investigators discovered two primary employment-related themes: 1) A lack of commitment or lack of job stability and 2) ongoing criminal behaviors to obtain money quickly. The latter behavior often led to the loss of existing employment opportunities.

#### 2.5.2 Posttraumatic stress symptoms

Trauma describes an overwhelming event that continues to have a negative impact on an individual long after the event has subsided, through nightmares, intrusive thoughts, hallucinations, and hypervigilance (54). Posttraumatic stress disorder (PTSD) occurs when these symptoms persist for at least one month. The rate of PTSD for the general American public is approximately 6.8% (55). Comparatively, male veterans of the Gulf War reported a rate of PTSD of 15.1%, and Vietnam War veterans reported a rate of 30.9%. However, 89% of individuals in commercial sexual exploitation in the United States met the criteria for a diagnosis of PTSD (56). The reported rate of PTSD for survivors of commercial sexual exploitation is higher than any other population exposed to trauma, including battered women, torture survivors, veterans, and victims of childhood trauma (2). Reduction of PTSD symptoms—along with the social and economic burden of PTSD—is a key marker for health and success for survivors of commercial sexual exploitation (2, 57–60).

#### 2.5.3 Sobriety

Substance abuse has long been suggested as a chief push factor for entry into commercial sexual exploitation (as in 50). According to Potterat et al. (3), drug- and alcohol-related causes of death were two of the leading causes of death for individuals in prostitution in the US, following homicide. Women in Young et al.’s (61) study stated that drugs assisted with increasing confidence, perceptions of control, and perceptions of closeness to others while decreasing feelings of guilt and sexual distress. Exploiters may manipulate and control individuals by giving or withholding substances (50, 62) or take advantage of pre-existing substance use disorders as a recruitment strategy (50, 63, 64).

Nonetheless, Farley et al. (2) found that less than half (48%) of the sample (*n* = 854) reported drug use, and 52% reported alcohol use. Several studies propose that substance abuse succeeded entry into commercial sexual exploitation for the majority of those who use substances (15, 61, 65–68), with only one suggesting the alternative (69). Even in Marshall’s (39) article, alcoholism was suggested as a secondary issue to that of poverty and food insecurity.

For those with substance use disorders, treatment and support challenges persist (59). Programs are rarely equipped to address trauma related to ACES, trauma related to commercial sexual exploitation, and co-occurring substance use disorders (62, 70, 71). Additional challenges are incurred when programs require sobriety and can evict individuals if they are caught using (71).

### 2.6 Length of time out of exploitation as a consideration

The period following one’s exit out of exploitation is a time when survivors may address vulnerabilities to exploitation and decrease the likelihood of revictimization. For example, survivors may go back to school, receive substance abuse treatment or job training, and pursue other economic empowerment opportunities. For this reason, the length of time one is out of commercial sexual exploitation should be considered in statistical models. Indeed, human trafficking researchers have begun to consider this period in their investigations, as in Edgemon et al. (72) and Clay-Warner et al. (73), who controlled for time since last exploitation for studies examining the effects of trafficking on women in Ghana.

### 2.7 The present study

The purpose of this study is to develop a deeper understanding of the lived experiences of survivors of commercial sexual exploitation by assessing the complex relationships between childhood background, trafficking experiences, and long-term outcomes. To that end, we analyzed data from a survey representing 350 adult survivors of commercial sexual exploitation living in the US. There are four aims associated with this investigation:

• Aim 1: Aim 1 will consider the relationship between childhood background and trafficking experiences (Model One).

o Hypothesis 1: Negative childhood experiences (i.e., higher number of ACES, higher socioeconomic disadvantage, and lower educational achievement) will be related to more negative trafficking experiences (i.e., earlier ages of entry into commercial sexual exploitation, longer experiences of commercial sexual exploitation, and higher number of negative trafficking experiences).

• Aim 2: Aim 2 will consider the relationship between trafficking experiences and survivor outcomes (Model Two).

o Hypothesis 2: Negative trafficking experiences (i.e., earlier ages of entry into commercial sexual exploitation, longer experiences of commercial sexual exploitation, and higher number of negative trafficking experiences) will be related to more negative outcomes (i.e., lower income, lower employment status, higher rate of PTSD symptoms, lower level of dignity, and lower rate of sobriety from drugs and alcohol).

• Aim 3: Aim 3 will consider the relationship between childhood background and survivor outcomes (Model Three).

o Hypothesis 3: Negative childhood experiences (i.e., higher number of ACES, higher socioeconomic disadvantage, and lower educational achievement) will be related to more negative outcomes (i.e., lower income, lower employment status, higher rate of PTSD symptoms, lower level of dignity, and lower rate of sobriety to drugs and alcohol).

• Aim 4: Aim 4 will consider the relationships between childhood background and outcomes and the indirect effects of trafficking experiences (Model Four).

o Hypothesis 4: There will be a significant indirect association between negative childhood experiences (i.e., higher number of ACES, higher socioeconomic disadvantage, and lower educational achievement), negative trafficking experiences (i.e., earlier ages of entry into commercial sexual exploitation, longer experiences of commercial sexual exploitation, and higher number of negative trafficking experiences), and more negative outcomes (i.e., lower income, lower employment status, higher rate of PTSD symptoms, lower level of dignity, and lower rate of sobriety to drugs and alcohol).

## 3 Methods

### 3.1 Sample description

**Figure 1:**
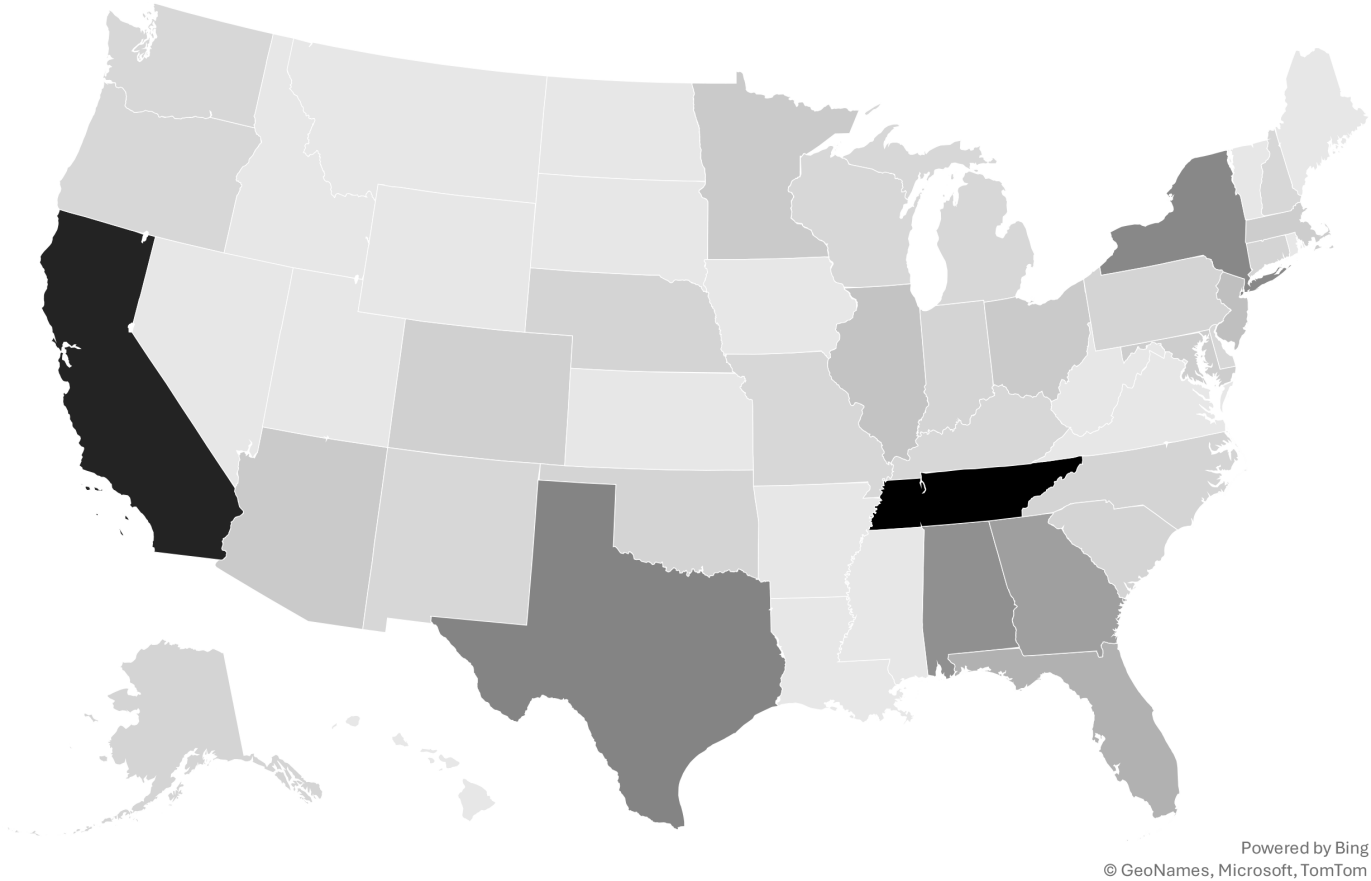
In which state of the United States do you currently live?

Individuals who were eligible to participate in the study (*n* = 350) were at least 18 years old, residents of the United States, and identified as survivors of commercial sexual exploitation. Participants were an average age of 32.61 years (min = 18.00, max = 73.00; *SD* = 9.99). Approximately 74.7% of study participants identified as female, 22.7% as male, 1.5% as genderqueer, 1.1% as gender-fluid, 1.1% as gender questioning, 1.1% as non-binary, 0.7% as gender non-conforming, 0.4% as agender, 0.4% as transgender female, and 0.4% as transgender male. The breakdown of race/ethnicity was as follows: American Indian/Alaskan Native (6.0%), Asian (0.8%), Black/African American (42.5%), Hispanic (8.6%), Middle Eastern (1.1%), Native Hawaiian (0.4%), White (46.9%), Multi-ethnic (7.5%), and another race not specified (1.1%). For gender and race/ethnicity, study participants were invited to mark all that applied. Respondents lived throughout the United States, with the majority living in Tennessee (19%), California (16.7%), Texas (8.9%), New York (8.6%), Alabama (7.8%), Georgia (6.3%), and Florida (4.5%; **Figure 1**).

### 3.2 Data collection procedures

Snowball sampling was used to recruit participants, as having survivors contact other survivors protected the privacy of all survivors and decreased potential coercion since they were not contacted by an “authority figure” whom they may or may not know personally (74, 75). Survivors who completed the survey were invited to share the link/QR code with their network of survivors of commercial sexual exploitation at the end of the survey. However, the participants were reminded that sharing information about the survey with others was not a requisite for participating in the research study (75). Participants who completed the survey received a $50 Visa gift card. Gift cards were mailed in blank thank-you notes to protect against third-party interception. We partnered with several direct-service organizations and professional networks to recruit research participants, including 4Sarah, BeLoved Atlanta, Family Sunshine Center, Frontline Response, the Global Association of Human Trafficking Scholars (GAHTS), the International Christian Alliance on Prostitution (ICAP), NightLight International, Rescuing Hope, Street Grace, and The WellHouse.

Data were collected via a self-directed, online survey facilitated by Qualtrics. A URL link and QR code directed potential participants to a detailed description of the study, which then linked to eligibility questions and the informed consent. Eligible participants were at least 18 years old, were residents of the United States, and self-identified as experiencing commercial sexual exploitation, defined as “Commercial sexual exploitation occurs when anything of value is given in exchange for a sex act.”

This community-based participatory (or survivor-informed) research (CBPR; 76–79) incorporated strengths-based language and used a trauma-informed framework (80). All survey measures, data collection procedures, and recruitment procedures were approved by a focus group of survivors of commercial sexual exploitation (i.e., lived experience experts). Focus group participants gave feedback on the appropriateness of questions, potential for misunderstandings, strengths-based language, and other opportunities to define and measure survivor perspectives and experiences. Once the focus group’s recommendations were addressed, the survey was piloted by another group of survivors of commercial sexual exploitation, whose feedback was also incorporated into the final version of the survey.

The trauma-informed survey consisted of 13 sections covering topics related to physical health, mental health, childhood experiences, relationships, service usage, and encounters with law enforcement. The survey took approximately 90 to 120 minutes. Participants were reminded to take breaks and skip any questions that they did not want to answer or select the option, “I prefer not to answer.” Additionally, Phonic was a widget approved by Auburn University that integrated into Qualtrics to collect voice-to-text responses, making it easier for participants with lower literacy and/or limited English proficiency to respond to questions in their own words. Survey fatigue was mitigated using skip logic such that only relevant questions were presented to each participant.

Funding for this project was provided by Auburn University’s College of Human Sciences, Auburn University’s Graduate School, the Department of Human Development and Family Science, the Margaret K. Keiley Scholarship, the Kappa Omicron Nu Maude Gilchrist Fellowship, and the Women's Philanthropy Board. The study was approved by the Auburn University Institutional Review Board (IRB) for protocol #24-898 MR 2406 effective as of July 1, 2024. The focus group and pilot projects were also conducted under the oversight of Auburn University's IRB (Protocol Review Request #23-133, “Sex trafficking prevalence, indicators, and survivor outcomes focus group;” Protocol Review #23-595, “Sex trafficking prevalence, indicators, and survivor outcomes”).

### 3.3 Measurements

#### 3.3.1 Childhood background

##### 3.3.1.1 Childhood socioeconomic disadvantage

A childhood socioeconomic disadvantage score was constructed using three indicators: Childhood welfare status (2 - ever on welfare, 0 - never on welfare), highest level of parental education (2 - less than high school, 1 - high school/GED, 0 - some college or higher), and financial level growing up (2 - worse off than others, 1 - about the same as others, 0 - better off than others). These scores were added together to represent childhood socioeconomic disadvantage such that higher scores indicated higher levels of disadvantage (81, 82).

##### 3.3.1.2 Adverse childhood experiences

Information about participants’ adverse childhood experiences (ACES) was collected utilizing the Adverse Childhood Experiences International Questionnaire (ACE-IQ; 83), which consisted of ten items. Participants were asked questions like, “Did a parent or adult in your home ever swear at you, insult you, or put you down?;” “Did a parent or adult in your home ever hit, beat, kick, or physically hurt you in any way?;” and “Did you experience unwanted sexual contact (such as fondling or oral/anal/vaginal intercourse/penetration)?” Each affirmative response was coded as 1. The Cronbach’s Alpha for the ACE-IQ was acceptable at.68. Scores on each item were added together to create a cumulative total risk score representing ACES.

##### 3.3.1.3 Education

Participants were asked, “What is the highest level of education you have completed?” Responses were ranked on an ordinal scale of “No schooling completed,” “Elementary school,” “Middle school,” “Some high school, no diploma,” “High school diploma or equivalent,” “Trade/technical/vocational training,” “Some college credit, no degree,” “Associate degree,” “Bachelor’s degree,” and “ Some post undergraduate work,” “Master’s degree,” “Specialist degree,” “Applied or professional doctorate degree,” “Doctorate degree,” and coded 0-13, respectively.

#### 3.3.2 Trafficking experiences

##### 3.3.2.1 Age of entry

Participants were asked, “How old were you the first time anyone gave you or someone else something of value (for example, money, shelter, clothing, food, toys, etc.) in exchange for a sex act with you (for example, intercourse, oral sex, anal sex, groping/fondling, stripping/exotic dancing, pornographic photos/videos, etc.)?” to determine age of entry into commercial sexual exploitation.

##### 3.3.2.2 Length of exploitation

To determine length of commercial sexual exploitation, participants were asked, “Using your best guess, what is the total amount of time you experienced commercial sexual exploitation (in other words, how long were you ‘in the life’)?” Responses were provided in days, weeks, months, and years. Length was then recoded to represent the total number of years in exploitation.

##### 3.3.2.3 Trafficking experiences checklist

The Trafficking Experiences Checklist is a novel instrument developed by the focus group of survivors of commercial sexual exploitation and the primary investigator. Study participants were asked, “While you were experiencing commercial sexual exploitation (in other words, in ‘the life’), did anyone ever do any of the following? (Please mark all that apply.)” Options included, “Physically hurt you,” “Harm your loved ones,” “Threaten you with a weapon,” and others. Responses were coded as Yes (coded as 1) or No (coded as 0). The Cronbach’s Alpha for the Trafficking Experiences Checklist was acceptable at.92. Numbers from the checklist were added together to create a cumulative risk score representing Trafficking Experiences.

#### 3.3.3 Outcomes

##### 3.3.3.1 Income

Participants were asked to provide their annual income from all sources, before taxes. Options ranged from “No income” (coded as 0) to “$100,000 or above” (coded as 11) with intervals of $9,999.00.

##### 3.3.3.2 Employment

Participants were asked to describe their employment situation. Options included employed full-time, employed part-time (i.e., 39 hours a week or less), self-employed, student, unemployed looking for work, unemployed not looking for work, homemaker/stay-at-home parent, unable to work, disability/supplemental security income (i.e., SSI/SSDI), and retired. To measure employment status, a new variable was created representing full-time employment (coded as 2), part-time employment (coded as 1), and unemployment (coded as 0).

##### 3.3.3.3 Posttraumatic stress disorder symptoms

The Posttraumatic Stress Disorder (PTSD) Checklist for *DSM-5* (PCL-5; 84) is a 20-item scale used to measure current PTSD symptoms. Participants were asked, “In the past month, how much were you bothered by…” Options included, “Repeated, disturbing, and unwanted memories of the stressful experience?,” “Repeated, disturbing dreams of the stressful experience?,” “Feeling very upset when something reminded you of the stressful experience?,” and others. Responses were ranked from “Not at all” to “Extremely,” and coded as 0 to 4. The Cronbach’s Alpha for the scale’s items was acceptable at.96. Responses to each item were added together and then divided by the total number of responses to create a total PTSD score that ranged between 0 and 4.

##### 3.3.3.4 Dignity

The Dignity Instrument, developed by Khatib and Armenian (85), is a 12-item scale used to determine participants’ level of worthiness, self-respect, self-esteem, and autonomy. On a five-point scale, where 0 = “Not at all,” and 4 = “Very much,” study participants were asked to rank statements like, “I feel peaceful,” “I have a reason for living,” and “My life has been productive,” and coded such that higher scores represented higher levels of dignity. The Cronbach’s Alpha for the Dignity Instrument’s scale items was acceptable at.85. Scores on each item were added together and then divided by the number of items to create one score representing “Dignity.”

##### 3.3.3.5 Substance use

###### 3.3.3.5.1 Alcohol use

The Alcohol Use Disorders Identification Test (AUDIT; 86) is a 10-item instrument measuring harmful alcohol consumption. On a 5-point scale ranging from “Never” to “Almost daily,” participants were asked to rate statements, like, “How often do you have a drink containing alcohol?,” “How often do you have six or more drinks on one occasion?,” and “How often during the last year have you failed to do what was normally expected of you because of drinking?” Scores were reverse coded, added together, and divided by the total number of responses to reflect an alcohol sobriety score, where 0 = “Extremely concerning alcohol use,” to 4 = “Low to no alcohol consumption.”

###### 3.3.3.5.2 Drug use

To measure illegal drug use in the past year, participants completed the Drug Abuse Screening Test (DAST-10; 87), a 10-item scale where participants were asked to rate statements, like, “Have you used drugs other than those required for medical reasons?,” “Do you use more than one drug at a time?,” and “Are you always able to stop using drugs when you want to?” Affirmative responses were coded as 1, added together, and then reverse coded to represent sobriety from illegal drugs.

#### 3.3.4 Control variable

Participants were asked, “How long ago was your last experience of commercial sexual exploitation? Possible responses included, “Within the past month,” “In the past 1–6 months,” “In the past 7–12 months,” “In the past 1–2 years,” “In the past 2–3 years,” “In the past 3–5 years,” “In the past 5–7 years,” “In the past 7–10 years,” and “More than 10 years ago,” to determine length since last experience of exploitation. Responses were coded 0 to 8. Length since last exploitation was used as a control variable.

### 3.4 Plan of analysis

#### 3.4.1 Preliminary analyses

The Statistical Package for Social Sciences (SPSS) version 29.0.2.0 was used to evaluate descriptive statistics, frequencies, and distributions of predictors and outcomes (88). Descriptive statistics included means, medians, modes, standard deviations, minimums, maximums, and ranges. SPSS was also used in bivariate correlation analyses.

##### 3.4.1.1 Missing data

As a trauma-informed approach to data collection, participants were allowed to skip any question they did not want to answer or respond with “Don’t know/Don’t remember” or “I prefer not to answer.” For this reason, it was expected that missing data needed to be addressed. First, missing data were coded as −99. Then, missing data were evaluated for patterns of missingness in two ways: 1) Visually, and 2) by creating a new variable representing the total number of missing variables for each case and conducting regression analyses to evaluate whether any of the dependent variables are related to the total amount of missing variables. There was only one significant pattern of missingness: Sobriety from drugs. This pattern of missingness is likely due to the skip logic function in that participants who responded that they never used drugs were not presented with irrelevant drug use-related questions. Finally, missing data were addressed using Full Information Maximum Likelihood estimation (FIML; 89).

#### 3.4.2 Primary analyses

Based on preliminary analyses of the mean, variance, and distribution of the outcome variable, we chose the most appropriate regression analyses to examine the relationship between childhood background and trafficking experiences (Aim 1), trafficking experiences to outcomes (Aim 2), childhood background and outcomes (Aim 3), and childhood background, trafficking experiences, and outcomes (Aim 4). Linear regression was used for the normally distributed, continuous dependent variables. Aim 4 evaluated the indirect effects of trafficking experiences on the links between childhood background and outcomes. Primary analyses were conducted using MPlus (90). Predictor variables were grand-mean-centered. Goodness of fit statistics were computed where appropriate, including root mean square error of approximation (RMSEA; 91), comparative fit index (CFI; 92), and Tucker-Lewis Index (TLI; 92).

## 4 Results

### 4.1 Preliminary analysis

#### 4.1.1 Childhood background

##### 4.1.1.1 Adverse childhood experiences

The mean number of ACES for study participants was 6, and 80% reported 4 or more ACES. The most common ACES were loss of a parent (via divorce or death), sexual abuse, and verbal abuse, respectively (**Table 1**).

**Table 1:** Results of ACES questionnaire.

Item text	%
Did you feel that you didn’t have enough to eat, had to wear dirty clothes, or had no one to protect or take care of you?	51
Did you lose a parent through divorce, abandonment, death, or other reason?	75
Did you live with anyone who was depressed, mentally ill, or attempted suicide?	50
Did you live with anyone who had a problem with drinking or using drugs, including prescription drugs?	62
Did your parents or adults in your home ever hit, punch, beat, or threaten to harm each other?	43
Did you live with anyone who went to jail or prison?	42
Did a parent or adult in your home ever swear at you, insult you, or put you down?	67
Did a parent or adult in your home ever hit, beat, kick, or physically hurt you in any way?	63
Did you feel that no one in your family loved you or thought you were special?	51
Did you experience unwanted sexual contact (such as fondling or oral/anal/vaginal intercourse/penetration)?	70

##### 4.1.1.2 Childhood socioeconomic disadvantage

More than half of respondents (57%) report utilizing some form of welfare during their childhood. Welfare programs in the US include food vouches (referred to as Electronic Benefits Transfer, EBT; or SNAP), subsidized housing (i.e., section 8 or Housing Choice Vouchers), the Women, Infants, and Children (WIC) Program, temporary financial aid programs, like Temporary Assistance for Needy Families (TANF), Childcare and Parent Services (CAPS), and others. Forty percent (40%) rated their financial status growing up as “Worse off than others,” and 42% rated their financial status, “About the same as others.” Participants described their mother’s highest level of education, where 12% had less than a high school diploma, 39% had a high school diploma or GED, and 50% had some college or higher. As to their father’s highest level of education, 16% had less than a high school diploma, 32% had a high school diploma or GED, and 51% had some college or higher.

##### 4.1.1.3 Educational achievement

The spread of educational achievement was such that 3.4% had no schooling, 0.8% only had an elementary school education, 4.2% had a middle school education, 16.7% had some high school, 21.6% had a high school diploma or General Educational Development diploma (GED), 4.5% went to a trade school, 12.5% had some college, 7.6% had an associate’s degree, 16.7% had a bachelor’s degree, 2.3% had some postgraduate credit, and 9.9% had a postgraduate degree.

#### 4.1.2 Trafficking experiences

**Figure 2:**
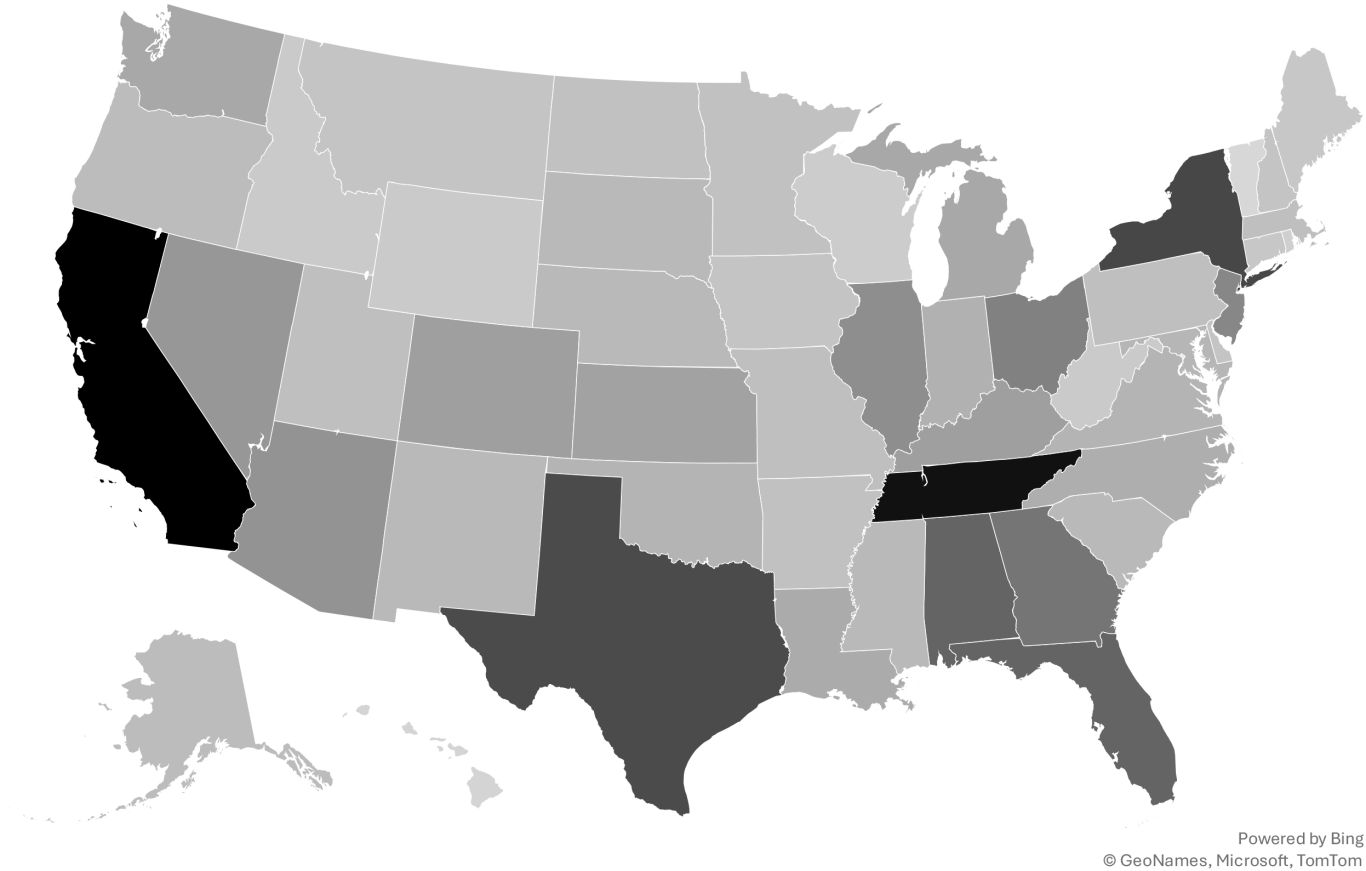
To your knowledge, in which state(s) did you experience commercial sexual exploitation? (Please mark all that apply.).

Participants report experiencing exploitation in all states excluding Vermont, with participants reporting the most experiences in California (18%), Tennessee (17%), New York (13%), Texas (13%), Alabama (11%), Florida (11%), Georgia (10%), Ohio (9%), New Jersey (8%), and Illinois (7%; **Figure 2**). The number of states in which each survivor experienced exploitation ranged from one state to 27 states, with a median of one state.

##### 4.1.2.1 Age of entry and length of exploitation

The mean age of entry into exploitation was 15 years old, with 70% entering exploitation before the age of 18 (*min* = 0, *max* = 50). Length of exploitation ranged from less than one year to fifty years, and the average length of exploitation was 9 years. The majority (94%) of participants’ exploitation experiences fall under the US federal definition of sex trafficking victimization.

##### 4.1.2.2 Trafficking experiences checklist

Participants used the Trafficking Experiences Checklist (**Table 2**) to list a variety of negative experiences that occurred while they were experiencing commercial sexual exploitation. The total number of negative trafficking experiences ranged from 1 to 36, with a mean of 11 negative trafficking-related experiences. The most common experiences included being made to feel scared or unsafe, being pressured or forced to touch someone or have any unwanted physical or sexual contact with another person, control of money/payment, control when and where they could go, being physically hurt, and being tricked into doing something they did not want to do. Less common, but concerning, experiences included being forced to get pregnant, being forced to get a tattoo/brand, being forced to participate in religious rituals/practices, withholding alcohol, and being forced to self-mutilate. Several participants added an experience in the “Other” option of being forced to participate in illegal activities.

**Table 2:** While you were experiencing commercial sexual exploitation (in other words, in “the life”), did anyone ever do any of the following?

Item text	%		%
Control how much food you ate	36	Make you do work or other activities that were different from what you were promised or told	33
Control the payment/money you should have been paid	52	Make you feel scared or unsafe	59
Control when you could sleep	37	Make you sign a document	20
Control where and/or when you could go places	48	Make you witness someone being harmed or threatened with harm	37
Control where you lived	33	Monitor you or stop you from calling family, friends, or others	44
Deprive you of sleep, food, water, or medical care	27	Physically hurt you (for example, beat, cut, slap, hit, kick, punch, burn)	48
Drug you without your knowledge	31	Pressure or force you to touch someone or have any unwanted physical or sexual contact with another person	50
Force you to drink alcohol	23	Take and/or keep your identification, for example, your passport or driver’s license	19
For you to engage in illegal activities*	1	Take a photo or video of you that you were uncomfortable with	43
Forced you to get an abortion	13	Take you out of school	12
Forced you to get a tattoo/brand	9	Tell you to lie about your age and/or the type of work you do	26
Force you to get pregnant	7	Threaten to harm your loved ones (for example, family, friends, pets, etc.)	30
Force you to get or use false identification or documentation	23	Threaten to hurt you (for example, beat, cut, slap, hit, kick, punch, burn)	53
Force you to participate in religious rituals/practices	10	Threaten to report you to the police or other authorities (for example, ICE, DHS, DCF, etc.)	20
Force you to self-mutilate	10	Threaten you with a weapon	38
Force you to take drugs	37	Trick or pressure you into doing something you did not want to do	46
Give your payment/money to someone else	30	Withhold alcohol from you	10
Harm your loved ones (for example, family, friends, pets, etc.)	14	Withhold payment/money from you	39
Lock you up, restrain you, or prevent you from leaving	35	Withhold drugs from you	22

*Item added by three participants in the “Other” field.

(Please mark all that apply.).

#### 4.1.3 Outcomes

##### 4.1.3.1 Income

Approximately 28% of research participants reported making less than $10,000.00 a year; 11% made between $10,000 and $19,000; 9% made between $20,000 and $29,000; 6% made between $30,000 and $39,000; 7% made between $40,000 and $49,000; 11% made between $50,000 and $59,000; 9% made between $60,000 and $69,000; 5% made between $70,000 and $79,000; 3% made between $80,000 and $89,000; 4% made between $90,000 and $99,000; and 7% made $100,000 or above.

##### 4.1.3.2 Employment

One-quarter of respondents were unemployed, 39.8% were employed part-time, and 35% were employed full-time. Approximately 11% were self-employed. Those who were not employed identified as student (5.1%), unemployed looking for work (11.4%), unemployed not looking for work (0.6%), homemaker/stay-at-home parent (1.4%), unable to work (5.7%), disability/supplemental security income (SSI/SSDI; 1.7%), and retired (0.6%).

##### 4.1.3.3 PTSD symptoms

Responses to the Posttraumatic Stress Disorder Checklist for *DSM-5* (PCL-5; 84) ranged from 0 to 4, with a mean value of 1.92 (**Table 3**). More than half (51.2%) of respondents currently meet the diagnostic criteria for a diagnosis of PTSD, and 76% have met the diagnostic criteria for PTSD at some point in their lives.

**Table 3:** Responses to the posttraumatic stress disorder checklist for DSM-5 (PCL-5; 84).

In the past month, how much were you bothered by…	*M*
Repeated, disturbing, and unwanted memories of the stressful experience?	1.92
Repeated, disturbing dreams of the stressful experience?	1.73
Suddenly feeling or acting as if the stressful experience were actually happening again (as if you were actually back there reliving it)?	1.51
Feeling very upset when something reminded you of the stressful experience?	1.95
Having strong physical reactions when something reminded you of the stressful experience (for example, heart pounding, trouble breathing, sweating)?	1.89
Avoiding memories, thoughts, or feelings related to the stressful experience?	2.13
Avoiding external reminders of the stressful experience (for example, people, places, conversations, activities, objects, or situations)?	2.14
Trouble remembering important parts of the stressful experience?	1.97
Having strong negative beliefs about yourself, other people, or the world (for example, having thoughts such as: I am bad, there is something seriously wrong with me, no one can be trusted, the world is completely dangerous)?	1.85
Blaming yourself or someone else for the stressful experience or what happened after it?	1.89
Having strong negative feelings such as fear, horror, anger, guilt, or shame?	2.04
Loss of interest in activities that you used to enjoy?	1.86
Feeling distant or cut off from other people?	1.98
Trouble experiencing positive feelings (for example, being unable to feel happiness or have loving feelings for people close to you)?	1.80
Irritable behavior, angry outbursts, or acting aggressively?	1.87
Taking too many risks or doing things that could cause you harm?	1.55
Being “super alert” or watchful or on guard?	2.30
Feeling jumpy or easily startled?	1.95
Having difficulty concentrating?	1.88
Trouble falling or staying asleep?	2.02

##### 4.1.3.4 Substance use

###### 4.1.3.4.1 Alcohol use

One-third (35%) of respondents report little to no alcohol consumption, and approximately one-quarter (24.3%) report non-problematic alcohol use. Only 15.1% report problematic alcohol consumption, and 3.7% report extremely concerning alcohol use.

###### 4.1.3.4.2 Drug use

Nearly three-fourths (74%) of respondents report illegal drug use in the past year, 41% report using more than one illegal drug, and 32% stated that they neglected their family because of illegal drug use. The median score was 6, and the data were skewed with a visible tail in the negative direction (*min* = 0.00; *max* = 10.00; *skew* = -.36; *se* = .19).

##### 4.1.3.5 Dignity

The mean score on the Dignity Instrument (85) was 2.77 (*min* = 0.42, *max* = 4.00, *SD* = 0.83), where 6.9% scored low on the scale, and 65.4% scored high (**Table 4**). These scores were lower than the population for which the instrument was developed (i.e., Palestinian refugees).

**Table 4:** Responses to dignity instrument.

Indicate how much these statements apply to you based on the past 7 days	*M*
I feel peaceful.	2.50
I have a reason for living.	3.09
My life has been productive.	2.80
I have trouble feeling peace of mind.	1.97
I feel a sense of purpose in my life.	2.76
I am able to reach down deep into myself for comfort.	2.48
I feel a sense of harmony within myself.	2.55
My life lacks meaning and purpose.	1.50
I find comfort in my faith or spiritual beliefs.	2.76
I find strength in my faith or spiritual beliefs.	2.76
Difficult times have strengthened my faith/spiritual beliefs.	2.71
Even during difficult times, I know that things will be okay.	2.76

#### 4.1.4 Control variable

*Length Since Last Exploitation.* On average, participants have been out of exploitation for 2 to 3 years. Approximately 14% of study participants report trading sexual activities for something of value within the previous year, 25% traded sex within the past 1 to 2 years, and 15% traded sex more than 10 years ago (**Table 5**).

**Table 5:** Length of time since last experience of commercial sexual exploitation.

Time since last experience of sexual exploitation	*M*
Within the past month	.061
In the past 1 to 6 months	.058
In the past 7 to 12 months	.020
In the past 1 to 2 years	.248
In the past 2 to 3 years	.143
In the past 3 to 5 years	.099
In the past 5 to 7 years	.133
In the past 7 to 10 years	.085
More than 10 years ago	.153

#### 4.1.5 Bivariate correlations

Childhood background variables (e.g., childhood socioeconomic background, ACES, and educational achievement) were strongly correlated with one another in the expected directions, as were trafficking experiences variables (e.g., age of entry, length of exploitation, and Trafficking Experiences Checklist). Outcome variables, income, employment status, and drug sobriety were linked in the expected directions, while dignity and alcohol sobriety were not as strongly linked to the other outcome variables. The control variable, longer length out of exploitation, was linked to higher educational achievement, lower levels of PTSD symptoms, higher employment status, and higher levels of sobriety from drugs (**Table 6**).

**Table 6:** Bivariate correlations.

Variable	ACES	Education	Welfare	Financial status	Mother education	Father education	Trafficking experiences	Length of exploitation	Age of entry	PTSD	Income	Employment	Dignity	ETOH sobriety	Drug sobriety	Length out
ACES	–	-.126*	.358***	.277***	.266***	.296***	.407***	.330***	-.136*	.166**	-.284***	-.094	.045	.107	-.079	.064
Education		–	-.125	.242***	-.174**	-.292***	-.004	-.193**	-.049	-.115	.406***	.413***	-.016	.019	.249***	.289***
Welfare			–	.231***	.200**	.341***	.157*	.108	-.099	.060	-.019	-.022	.022	.065	-.029	-.009
Financial Status				–	.362***	.297***	.124*	.060	-.055	.167**	-.304***	-.238***	-.095	.066	-.061	.058
Mother Education					–	.557***	.139*	.211**	-.094	-.119	-.259***	-.096	.020	.052	.034	.027
Father Education						–	.142*	.213**	-.110	-.032	-.264***	-.084	.131	.049	-.066	.030
Trafficking Experiences							–	.272***	-.136*	.062	-.222***	-.115	.075	.063	.170*	.120*
Length of Exploitation								–	-.182**	.015	-.165*	-.108	.015	.204***	-.086	.043
Age of Entry									–	.025	-.124	-.102	-.048	-.062	-.104	-.062
PTSD										–	-.161*	-.238***	-.230***	-.028	-.370***	-.290***
Income											–	.594***	-.022	.011	.254***	.157*
Employment												–	.140*	.053	.311***	.276***
Dignity													–	-.007	.151*	.092
ETOH Sobriety														–	.008	-.048
Drug Sobriety															–	.443***
Length Out																–

*Correlation is significant at the.05 level (2-tailed).

**Correlation is significant at the.01 level (2-tailed).

***Correlation is significant at the.001 level (2-tailed).

### 4.2 Primary analysis

Factor loadings for the latent variable representing childhood socioeconomic disadvantage were mixed (*λ*_Father_Education_ = .79, *λ*_Mother_Education_ = .72, *λ*_Welfare_ = .38, and *λ*_Financial_Status_ = 0.46). Model fit indices were generally acceptable (**Table 7**).

**Table 7:** Factor loadings for latent variable: childhood socioeconomic disadvantage.

Childhood Socioeconomic Disadvantage				
Indicator	Estimate	Standard error	Standard latent variable	*p*-value
Ever on welfare	.383	.069	5.582	.000
Financial level growing up	.455	.067	6.821	.000
Mother education	.717	.065	11.080	.000
Father education	.791	.065	12.181	.000
*X*^2^	7.106			
Degrees of freedom	2			
*p*-value	.0286			
CFI	.959			
TLI	.876			
RMSEA	.098			
SRMR	.032			

Results reported are standardized.

#### 4.2.1 Aim 1: childhood background and trafficking experiences

**Figure 3:**
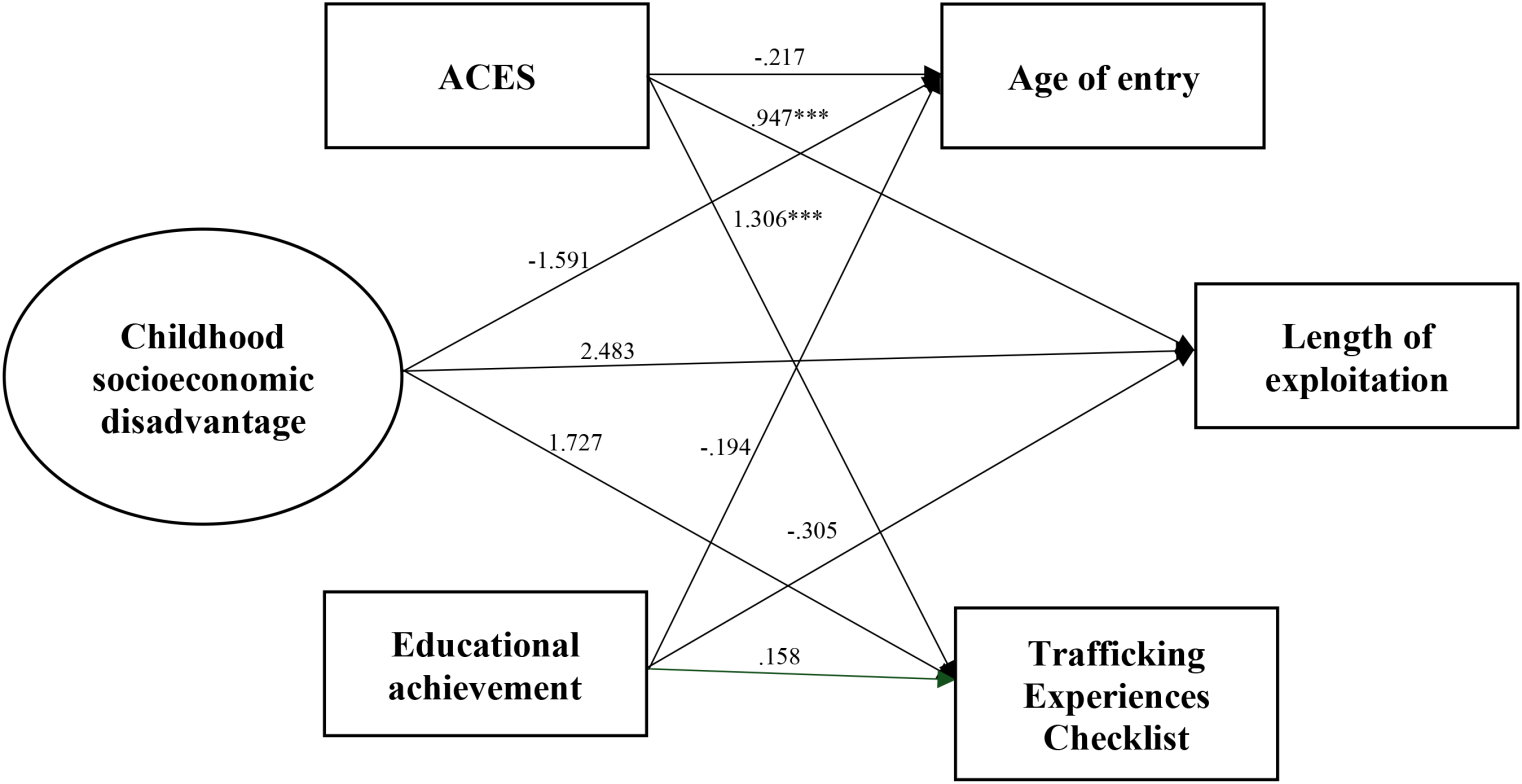
Model one: childhood background and trafficking experiences. Results reported are unstandardized. ***Correlation is significant at the 0.001 level (2-tailed).

To address Aim 1 of this investigation, a regression analysis was conducted to identify the links between childhood background and trafficking experiences (Model One; **Figure 3**). Goodness of fit statistics for Model One were acceptable (**Table 8**). Results indicated a significant positive link between ACES and the Trafficking Experiences Checklist (*B* = 1.31, *p* <.001) and length of commercial sexual exploitation (*B* = .95, *p* <.001), suggesting that increased number of ACES is associated with more severe trafficking experiences. Interestingly, there were no significant links between childhood socioeconomic disadvantage or educational achievement and trafficking experiences. Model One accounts for 19% of the variance for the Trafficking Experiences Checklist and 14% of the variance for length of commercial sexual exploitation (**Table 9**).

**Table 8:** Fit statistics for models one, three, and four.

Fit statistic	Model 1	Model 3	Model 4
*X* ^2^	35.553	78.236	98.886
Degrees of freedom	17	29	44
*p*-value	.005	.000	.000
CFI	.933	.905	.913
TLI	.863	.755	.770
RMSEA	.057	.070	.060
SRMR	.039	.046	.043

Measures of model fit were: *X*^2^ = chi-square; *p*-value (<.05 indicates good model fit); CFI, comparative fit index (>.95 indicates good fit; 92); TLI, Tucker-Lewis index (>.95 indicates good fit; 92); RMSEA, root mean square error of approximation (<.05 indicates good fit; 91); SRMR, standardized root mean square residual (<.08 indicates good fit).

**Table 9:** Model one results for outcome variables, age of entry, length of exploitation, and the trafficking experiences checklist.

Predictor variable	Age of entry	Length of exploitation	Trafficking experiences checklist
ACES	-.217	.947***	1.306***
Educational achievement	-.194	-.305	.158
Childhood socioeconomic disadvantage	-1.951	2.483	1.727
*R*-squared	.034	.142***	.185***

Results reported are unstandardized.

***Indicates significance at the.001 level.

#### 4.2.2 Aim 2: trafficking experiences and outcomes

**Figure 4:**
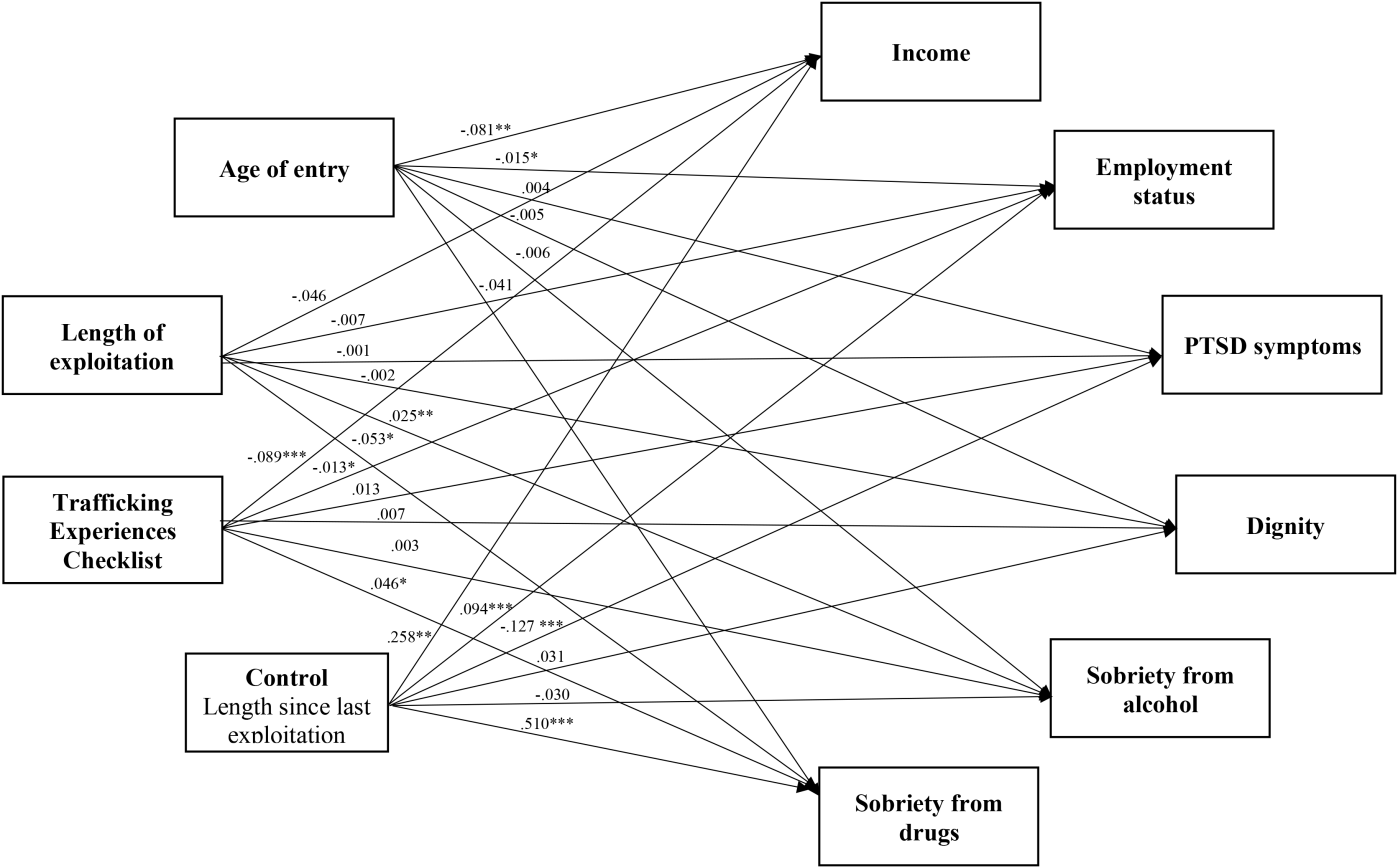
Model two: trafficking experience to outcomes with control variable, length since last exploitation. Results reported are standardized. *Correlation is significant at the 0.05 level (2-tailed). **Correlation is significant at the 0.01 level (2-tailed). ***Correlation is significant at the 0.001 level (2-tailed).

A linear regression model was conducted to address Aim 2 of this investigation and identify the links between trafficking experiences and outcomes for survivors of commercial sexual exploitation (Model Two; **Figure 4**). The model was just identified (i.e., zero degrees of freedom). Thus, model fit indices are not interpretable for Model Two. Results indicated that age of entry into commercial sexual exploitation had a small but significant negative link to income (*B* = -.081, *p* = .006) and employment status (*B* = -.02, *p* = .046). Length of commercial sexual exploitation also had small but significant negative links to sobriety from drugs (*B* = -.05, *p* = .023) and alcohol (*B* = .03, *p* = .002). The Trafficking Experiences Checklist was significantly related to income (*B* = -.09, *p* = .001), employment (*B* = -.01, *p* = .019), and sobriety from drugs (*B* = .05, *p* <.038). The control variable, length of time out of commercial sexual exploitation, was significantly linked to PTSD (*B* = -.13, *p* <.001), income (*B* = .26, *p* = .003), employment (*B* = .09, *p* <.001), and sobriety from drugs (*B* = .52, *p* <.001). Model Two accounts for 9% of the variance in PTSD symptoms, 11% of the variance in income and employment, and 20% of the variance in sobriety from drugs (**Table 10**).

**Table 10:** Model two results for outcome variables, income, employment, PTSD symptoms, dignity, and sobriety from drugs and alcohol with control variable, length since last exploitation.

Predictor variable	Income	Employment status	PTSD symptoms	Dignity	Sobriety from alcohol	Sobriety from drugs
Age of entry	-.081**	-.015*	.004	-.005	-.006	-.041
Length of exploitation	-.046	-.007	.001	-.002	.025**	-.053*
Trafficking experiences checklist	-.089***	-.013*	.013	.007	.003	.046*
Length since last exploitation	.258**	.094***	-.127***	.031	-.030	.510***
*R*-squared	.109**	.110**	.093**	.015	.040	.204***

Results reported are unstandardized.

*Indicates significance at the.05 level.

**Indicates significance at the.01 level.

***Indicates significance at the.001 level.

#### 4.2.3 Aim 3: childhood background and outcomes

**Figure 5:**
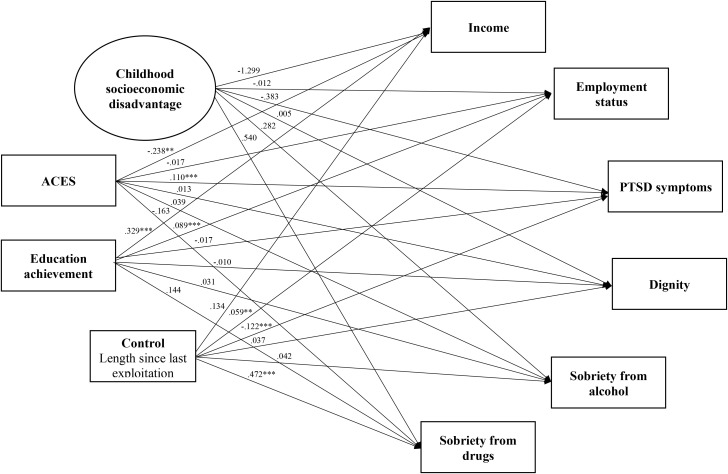
Model three: childhood background and outcomes with control variable, length since last exploitation.

To address Aim 3, a linear regression model was conducted to identify the links between childhood background and outcomes for survivors of commercial sexual exploitation (Model Three; **Figure 5**). Goodness of fit statistics for Model Three were acceptable (**Table 7**). Results suggested that increased ACES were linked to increased PTSD symptoms (*B* = .11, *p* <.001) and lower income (*B* = -.24, *p* = .008). Further, educational achievement was significantly linked to improved employment status (*B* = .09, *p* <.001) and increased income (*B* = .33, *p* <.001). Again, there were no significant links between childhood socioeconomic disadvantage and outcomes. The control variable, length of time out of commercial sexual exploitation, was significantly linked to PTSD (*B* = -.12, *p* <.001), employment (*B* = .06, *p* = .003), and sobriety from drugs (*B* = .47, *p* <.001). Model Three accounted for 14% of the variance in PTSD symptoms, 24% of the variance in income, and 20% of the variance in employment and sobriety from drugs (**Table 11**).

**Table 11:** Model three results for outcome variables, income, employment, PTSD symptoms, dignity, and sobriety from drugs and alcohol with control variable, length since last exploitation.

Predictor variable	Income	Employment status	PTSD symptoms	Dignity	Sobriety from alcohol	Sobriety from drugs
ACES	-.238**	-.017	.110***	.013	.039	-.163
Educational achievement	.329***	.089***	-.017	-.010	.031	.144
Childhood socioeconomic disadvantage	-1.299	-.012	-.383	.005	.282	.540
Length since last exploitation	.134	.059**	-.122***	.037	-.042	.472***
*R*-squared	.240***	.200***	.144**	.012	.023	.196***

Results reported are unstandardized.

**Indicates significance at the.01 level.

***Indicates significance at the.001 level.

#### 4.2.4 Aim 4: childhood background, trafficking experiences, and outcomes

**Figure 6:**
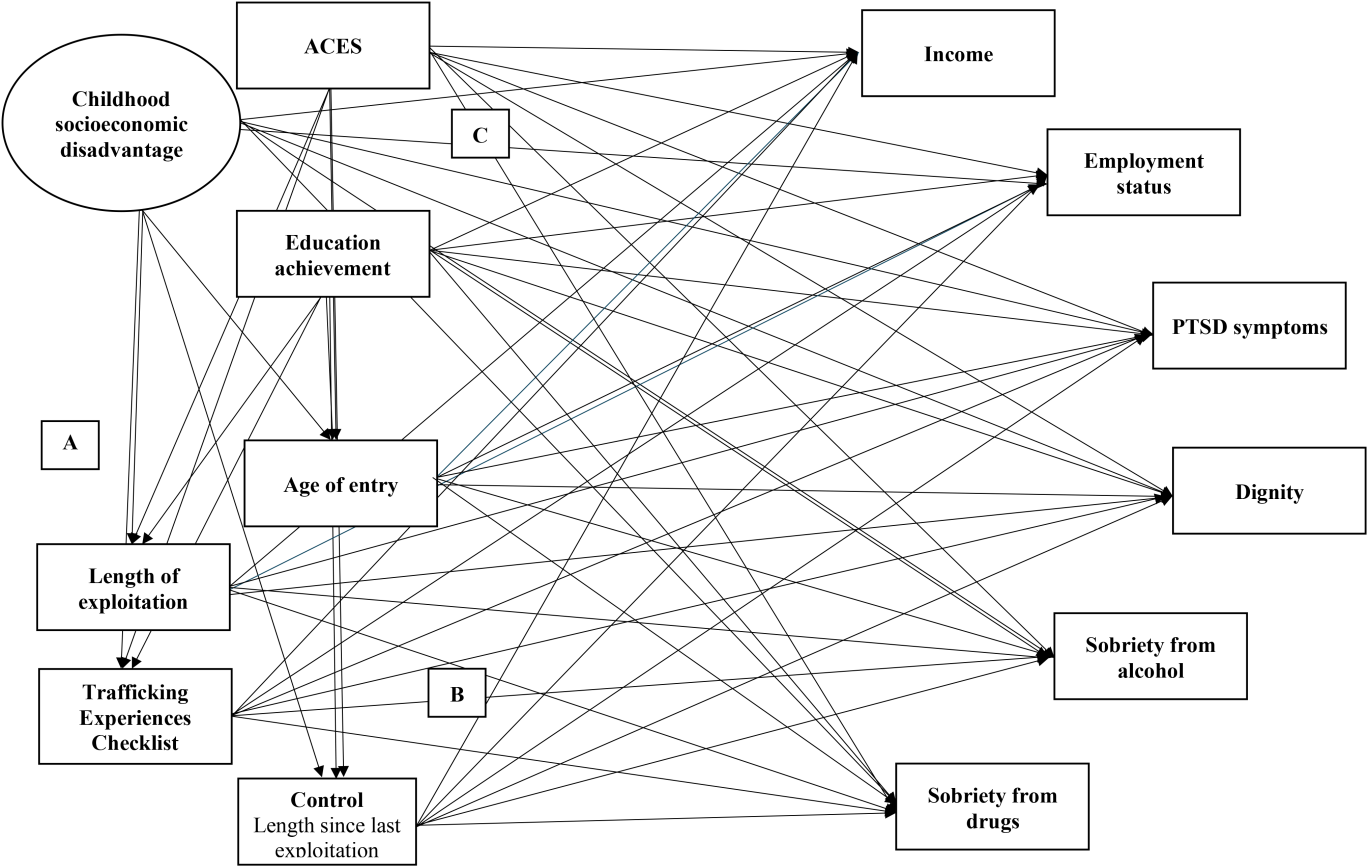
Model four: childhood background to trafficking experiences to outcomes with control variable, length since last exploitation.

**Figure 7:**
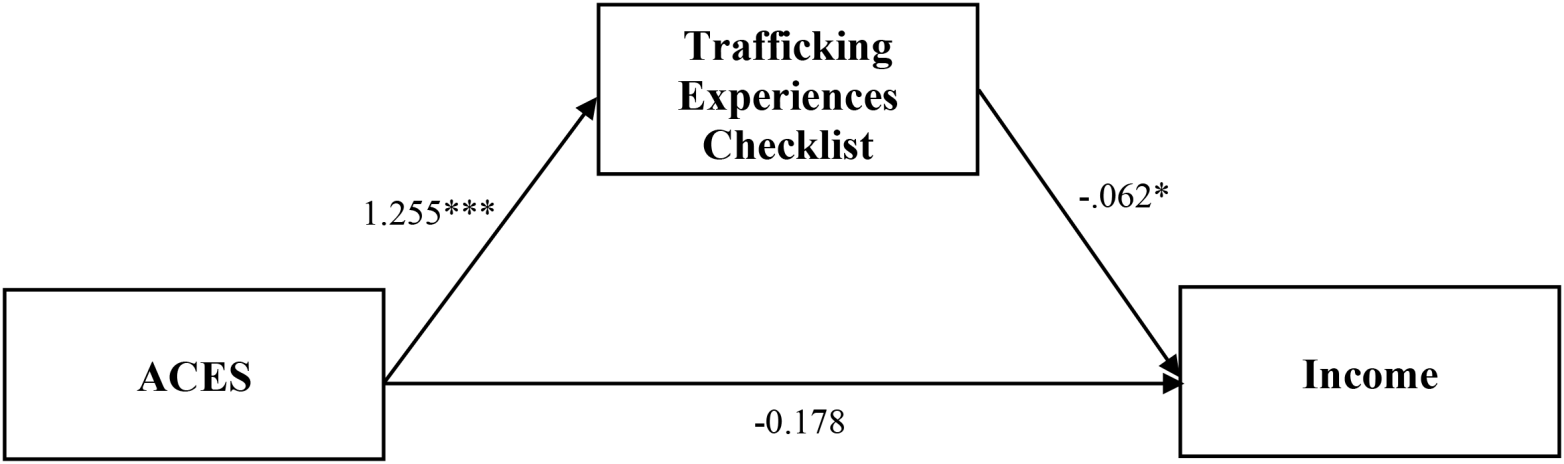
Analysis model of direct and indirect effects between ACES and income via the trafficking experiences checklist. Indirect effect equals -0.078*. *Coefficient is significant at the 0.05 level (2-tailed). ** Coefficient is significant at the 0.01 level (2-tailed). *** Coefficient is significant at the 0.001 level (2-tailed).

**Figure 8:**
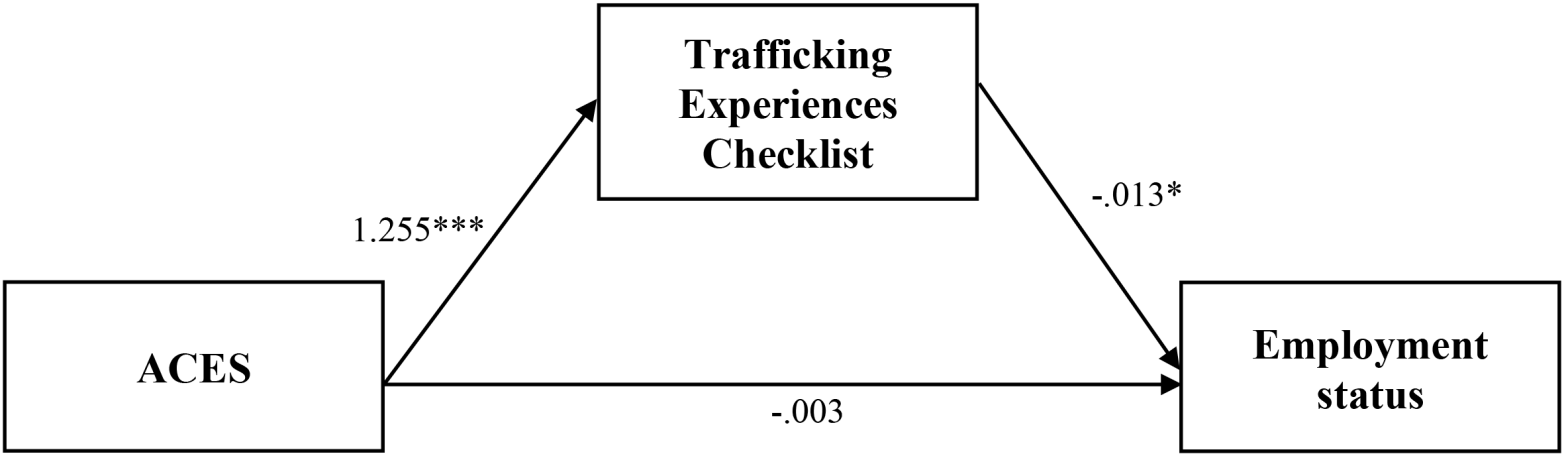
Analysis model of direct and indirect effects between ACES and employment via the trafficking experiences checklist. Indirect effect equals -0.016*. *Coefficient is significant at the 0.05 level (2-tailed). ** Coefficient is significant at the 0.01 level (2-tailed). *** Coefficient is significant at the 0.001 level (2-tailed).

**Figure 9:**
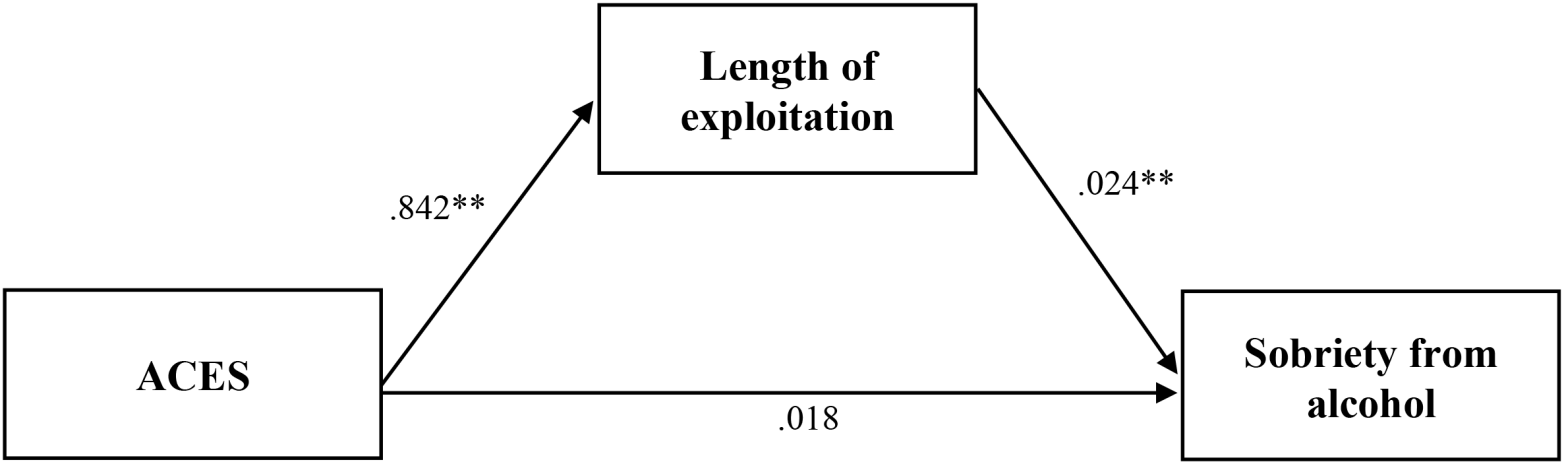
Analysis model of direct and indirect effects between ACES and sobriety from alcohol via length of exploitation. Indirect effect equals 0.021*. *Coefficient is significant at the 0.05 level (2-tailed). ** Coefficient is significant at the 0.01 level (2-tailed). *** Coefficient is significant at the 0.001 level (2-tailed).

**Figure 10:**
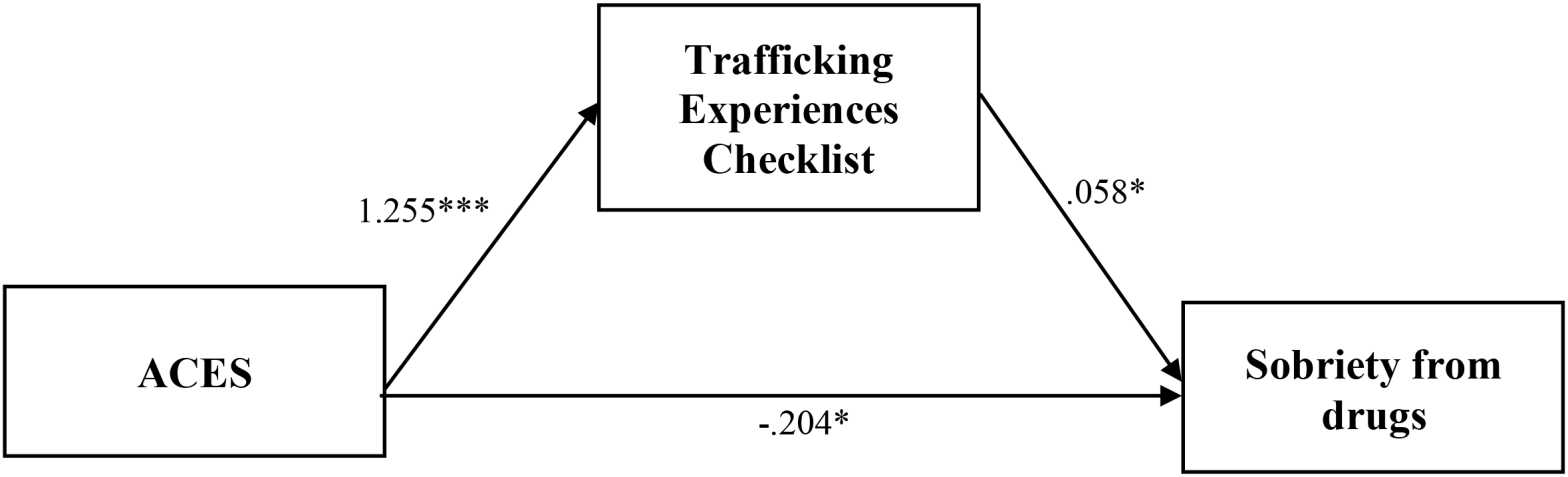
Analysis model of direct and indirect effects between ACES and sobriety from drugs via sobriety from drugs. Indirect effect equals 0.073*. *Coefficient is significant at the 0.05 level (2-tailed). ** Coefficient is significant at the 0.01 level (2-tailed). *** Coefficient is significant at the 0.001 level (2-tailed).

A mediation model was conducted to identify the links between childhood background and outcomes via trafficking experiences for survivors of commercial sexual exploitation for Aim 4 (Model Four; **Figure 6**). Goodness of fit statistics for Model Four were acceptable (**Table 7**). Income and employment were significantly indirectly linked to ACES through the Trafficking Experiences Checklist (*B* = -.08, p =.027; B = -.02, *p* = .046; **Figures 7** and **8**, respectively). Sobriety from alcohol was significantly linked to ACES through length of commercial sexual exploitation (*B = .*02, *p* = .032; **Figure 9**). Similarly, sobriety from drugs was linked to ACES through the Trafficking Experiences Checklist (*B = .*07, *p* = .024; **Figure 10**).

The following formula was used to determine the proportion of the indirect effect sizes:


$$Indirect\;effect\;size=\frac{Indirect\;effect}{Total\;effect}=\frac{ab}{ab+c'}$$

Overall, the proportion of the total effect of the *a, b*, and *c’* pathways between ACES and income through the Trafficking Experiences Checklist accounts for 30% of the total effect. The proportion of the total effect of the *a, b*, and *c’* pathways between ACES and employment through the Trafficking Experiences Checklist accounts for 84% of the total effect. The proportion of the total effect of the *a, b*, and *c’* pathways between ACES and sobriety from alcohol through length of commercial sexual exploitation accounts for 53% of the total effect. The proportion of the total effect between ACES and sobriety from drugs through the Trafficking Experiences Checklist accounts for 56% of the total effect.

## 5 Discussion

To develop a deeper understanding of the lived experiences of survivors of commercial sexual exploitation and determine the links between childhood background, trafficking experiences, and long-term outcomes, 350 adult survivors of commercial sexual exploitation living in the US were surveyed. Preliminary results of this investigation suggest that 94% of individuals who have experienced commercial sexual exploitation are victims of sex trafficking according to the US federal definition (22 U.S.C. § 7102). Interestingly, exploitation was identified in every state except Vermont. Common trafficking experiences included being made to feel scared or unsafe, being pressured to have unwanted contact with another person, loss of control over one’s agency and finances, physical abuse, and being tricked to do things they did not want to do. A small but concerning portion of the sample reported being forced to get pregnant, get a tattoo, participate in religious rituals, and self-mutilate. There were also reports of the potentially life-threatening practice of withholding alcohol.

Aims 1 through 3 considered the links between early childhood experiences (e.g., ACES, educational achievement, and childhood socioeconomic disadvantage), trafficking experiences (e.g., age of entry and length), and outcomes (e.g., income, employment status, PTSD symptoms, dignity, and sobriety). Survivors of commercial sexual exploitation report significantly more ACES than the general population, with 80% reporting 4 or more ACES. ACES were significantly linked to longer experiences of exploitation, more negative trafficking experiences, lower income, and increased PTSD symptoms. As expected, educational achievement was linked to increased income and employment status. However, educational achievement did not decrease the severity of trafficking experiences, even when controlling for length since last exploitation, contradicting previous findings (6). As in Potter et al. (28), childhood socioeconomic disadvantage was not significantly linked to any of the outcomes, suggesting that higher socioeconomic status is not protective against severe exploitation or its negative impact. This finding also contradicts much of the literature (as in 37–39, 93).

Longer experiences of exploitation were slightly linked to increased drug use, and the Trafficking Experiences Checklist was marginally associated with decreased income and employment status. Earlier ages of entry were somewhat associated with lower income and employment status, while longer experiences of exploitation were marginally linked with sobriety from alcohol. Similarly, trafficking experiences were positively linked to decreased drug use. This unexpected finding may be a result of posttraumatic growth or other resilience-building effects. Other possible reasons for the finding may include survivors’ engagement in their own recovery, motivation for change, or a protective practice, as using substances can increase vulnerability to abuse and exploitation. See section, Theoretical Connections, for more on this. Another surprising finding was that there was a weak positive association between the length of exploitation and sobriety from alcohol. This may be due to low disclosure of alcohol use among respondents. Interestingly, none of the predictors were linked to the outcome, dignity.

Aim 4 investigated the indirect pathways between early childhood experiences, trafficking experiences, and outcomes for survivors of commercial sexual exploitation. Results suggested that trafficking-related experiences may play a mediating role in the negative effects of early adversity on income and employment. Over half of the total effect of childhood adversity on alcohol sobriety outcomes among individuals can be explained by how long they were commercially sexually exploited. This suggests that longer exploitation, which is more likely with higher ACES, may reduce the likelihood of sobriety and that both factors are important predictors of sobriety outcomes. While higher number of ACES may have a negative overall effect on drug sobriety, the pathway through trafficking experiences reverses or dampens that effect, suggesting that trafficking experiences may improve the potential for sobriety that might not otherwise be associated with ACES. This finding suggests that individuals with trafficking experiences may have greater access to services, support systems, or interventions that promote sobriety, despite their high adversity. On the other hand, the findings may again represent posttraumatic growth or other resilience-building effects. See section, Theoretical Connections, for more on posttraumatic growth.

### 5.1 Theoretical connections

The study’s findings can be meaningfully interpreted through the lenses of both Bronfenbrenner’s Ecological Systems Theory (12) and Elder’s Life Course Theory (13). Considerations at multiple levels of the ecosystem (12) were included in the investigation, as well as concepts related to Life Course Theory, like timing (of exploitation), cumulative disadvantage, turning points, linked lives, and human agency (13). Together, it can be determined that development is dynamic and context-dependent. In other words, early adversity (microsystem; 12) and life transitions (chronosystem, 12; life course timing, 13) interact to shape outcomes.

Findings of this study also suggest that survivors may experience posttraumatic growth despite early, complex, cumulative trauma (94). Posttraumatic Growth is a theory that describes the positive changes that can occur at an individual level as a result of adversity and trauma. The experiences of posttraumatic growth incorporate new possibilities, a new appreciation for life, meaningful interpersonal relationships, a deeper spiritual life, and increased inner strength and confidence (95). Posttraumatic growth does not imply that the trauma was beneficial or desirable, and it can coexist with distress, grief, and symptoms related to PTSD. Posttraumatic growth is encouraged when individuals have access to trauma-informed support services, have supportive interpersonal relationships, and can make meaning out of negative circumstances. Evidence of posttraumatic growth might be seen by certain findings from this study, such as longer experiences of exploitation being linked with sobriety from alcohol, and more negative trafficking experiences being related to decreased drug use. Together, these findings align with theoretical models emphasizing human agency and meaning-making across the life course, providing a conceptual foundation for the applied implications discussed below.

### 5.2 Strengths

Due to ethical issues, as well as the criminal and covert nature of the crime of sex trafficking, survivors of commercial sexual exploitation are a notoriously difficult population for researchers to reach. Consequently, most studies are qualitative, consist of small sample sizes, and focus on minor sex trafficking (11, 14, 17, 96). For this reason, there are several strengths associated with this study. First, the study utilized a sample size that is substantial for this population and includes a good proportion of male respondents. Second, the comprehensive, trauma-informed survey provided an opportunity for us to quantitatively consider the link between several variables on outcomes, reflecting the dynamic lives of survivors of commercial sexual exploitation.

Next, the Trafficking Experiences Checklist, a novel scale developed by the focus group of survivors of commercial sexual exploitation and the primary investigator, was used to consider the effects of trafficking experiences on survivor outcomes. Similar to the ACES framework, future research could further develop and validate the Trafficking Experiences Checklist as a cumulative risk scale to better capture the complexity of exploitation experiences. An additional strength of this investigation is the use of behavioral descriptions rather than labeling descriptions of trafficking experiences. For example, study participants were asked, “At any time, did you ever feel forced (by violence or threat of violence to yourself or someone/something else) to trade something of value for a sex act (for example, intercourse, oral sex, anal sex, groping/fondling, stripping/exotic dancing, etc.)?” instead of, “Were you ever sex trafficked?” (97). A best practice for practitioners working with survivors already (98), these questions yielded an increase in disclosures of trafficking victimization, resulting in one of the highest and most accurate prevalence rates of trafficking victimization in the US.

The unique use of length since last exploitation as a control variable is another strength of this investigation. Length since last exploitation was strongly significantly linked to increased income and employment status, decreased PTSD symptoms, and increased sobriety from drugs, indicating that outcomes for survivors can improve over time, regardless of their background. This finding, along with others, suggests certain implications for practitioners and survivors.

### 5.3 Implications

The investigation provides insight into the lived experiences of survivors of commercial sexual exploitation, including their childhood background, trafficking experiences, and outcomes. Primarily, the investigation reveals that support services should be trauma-informed and consider the effects of childhood adversity, along with the negative experiences associated with exploitation. However, a “one-size-fits-all” approach is less than ideal for this population (99). Instead, services should be variable and address the unique needs of each individual. A simple intake checklist could help to identify the specific needs of an individual and assist survivors and practitioners in determining the best services or program plan for them.

Although time since last exploitation was associated with more favorable outcomes, it did not fully offset the effects of childhood adversity and trafficking experiences. Accounting for time since exploitation helped clarify these relationships and may assist practitioners in developing both short- and long-term support strategies. Another promising implication of this study is that individual dignity may be supported regardless of background or exploitation history. Although dignity was not significantly associated with the predictors, this finding may suggest that an internal sense of worth is relatively resilient or that existing measures may not fully capture how dignity is experienced among survivors. Research into the measurement and construct validity of the measure of dignity for this population is a critical future direction. Regardless, the finding underscores the importance of dignity as a strengths-based focus of survivor-centered services.

In addition, findings related to substance use suggest implications for addiction treatment programs working with survivors of commercial sexual exploitation. The association between longer exploitation histories and increased sobriety from alcohol may reflect opportunities for posttraumatic growth, particularly when survivors are supported in meaning-making and recovery-oriented processes. Addiction treatment approaches that are trauma-informed and strengths-based may benefit from acknowledging survivors’ adaptive coping, resilience, and agency while remaining attentive to ongoing distress and relapse risk. Emphasizing survivor-defined goals, peer support, and narrative-based interventions may help treatment providers leverage survivors’ existing capacity for growth without framing trauma as beneficial or necessary.

The last implication suggests that behavioral descriptions rather than labeling descriptions should be used with survivors of commercial sexual exploitation, as they yield more disclosure of vulnerability and victimization (97, 98). The focus group also suggested that providing a list of examples could help survivors better recognize their own experiences and potentially increase disclosure rates. These implications can be broadly applied within the anti-trafficking movement as we address the complex and multifaceted phenomenon of commercial sexual exploitation from within our various spheres of influence.

### 5.4 Limitations

This investigation addressed several gaps in the literature. Even so, there are several limitations related to this study. First, the use of subjective, self-report, retrospective data makes it difficult to confirm the veracity of some responses. Also, the study did not include a “control group” (i.e., a group who did not experience commercial sexual exploitation), rendering between-group analyses unavailable. Even so, within-group analyses are helpful to survivors, practitioners, and policymakers alike.

The sample also demonstrated substantially higher educational achievement relative to other datasets representing survivors of commercial sexual exploitation (e.g., 6). Several factors may have contributed to this pattern, including snowball sampling, survey accessibility barriers related to literacy, English proficiency, or Internet access, and selection effects whereby individuals with higher educational achievement may be more likely to participate in research. Elevated educational achievement may shape access to resources, coping strategies, help-seeking behaviors, and post-exit opportunities, potentially influencing observed associations among study variables. Consequently, some survivors—particularly those with lower educational achievement—may be underrepresented in the present investigation. Practitioners and service providers should therefore interpret findings with caution when working with survivors who have limited formal education, as intervention needs, resource accessibility, and pathways to recovery may differ from those reflected in this sample.

The latent construct representing childhood socioeconomic disadvantage included multiple ordinal indicators and a dichotomous indicator of welfare receipt. Mixed-scale indicators often produce weaker loadings and poorer global fit indices because they introduce distributional heterogeneity into the measurement model. In our analyses, the welfare indicator loaded in the expected direction, and the structural paths remained stable, suggesting that these measurement constraints reflect model complexity rather than substantive misspecification. Given the conceptual importance of welfare exposure for capturing childhood poverty, the indicator was retained, and results should be interpreted with appropriate caution.

While we attempted to include a diverse collection of variables in the models, many other factors that were not included in the models could play a role in producing more positive outcomes for survivors of commercial sexual exploitation. Finally, the investigation was only open to those residing in the US. Likewise, snowball sampling limited the survey’s reach. Therefore, caution should be given as to the generalizability of the findings for other geographical or cultural contexts.

### 5.5 Future research

Future research into the experiences of and responses to commercial sexual exploitation would benefit from building a more comprehensive understanding using large sample sizes and data collected across multiple time points (i.e., longitudinal designs). In particular, future studies should prioritize analytic approaches that allow for the disaggregation of data by race and gender, as survivor and focus group feedback emphasized meaningful differences in experiences and support needs among groups such as Black survivors and male survivors (100). Such approaches are essential for developing specialized and culturally responsive supports rather than assuming uniform intervention needs across survivor populations.

Along with subjective data, objective measures should be incorporated and include a range of indicators such as social determinants of health. The Trafficking Experiences Checklist could be strengthened by incorporating a severity scale, capturing the frequency of negative experiences, or integrating structured qualitative data to provide a more nuanced depiction of trafficking experiences. To improve the measurement of childhood socioeconomic disadvantage, future research should consider operationalizing welfare exposure as an ordinal rather than dichotomous variable to allow for greater variability and improved model fit.

A natural extension of this work would be to examine whether specific support services, their perceived helpfulness, or relationships with caregivers and other social supports are stronger predictors of positive outcomes. Such studies should continue to control for time since last exploitation to isolate developmental and contextual effects. Posttraumatic growth measures, such as the Posttraumatic Growth Inventory (PTGI; 101), may be particularly useful for expanding upon the unexpected findings observed in this investigation. As research in this area continues to evolve, disaggregated and methodologically rigorous approaches will better equip stakeholders to address structural vulnerability, support survivor recovery, and prevent future exploitation.

## 6 Conclusion

The purpose of this investigation was to consider the relationship between childhood background, commercial sexual exploitation experiences, and outcomes for adult survivors (n = 350) living in the US. Preliminary results suggested that a high rate of survivors report four or more ACES and come from mixed socioeconomic backgrounds. Overall, 94% of those surveyed reported experiences in alignment with the US federal definition of sex trafficking victimization. The most common trafficking experiences included being made to feel scared, loss of control of their money, and being forced to touch someone physically or sexually.

Primary analyses went on to reveal that adverse childhood experiences were linked to more negative trafficking experiences, though childhood socioeconomic background and educational achievement were not. A variety of factors, like low educational achievement, earlier ages of entry into commercial sexual exploitation, and negative trafficking experiences, were linked to lower income and employment status, but findings related to sobriety were mixed. In some cases, more adversity was linked to more positive outcomes, perhaps due to posttraumatic growth or other resilience-building effects. This investigation highlights the diverse and individualized needs of survivors of commercial sexual exploitation, indicating that tailored interventions are essential for addressing the complex interplay of childhood adversity, trafficking experiences, and recovery trajectories.

## Data Availability

The datasets presented in this article are not readily available because they contain sensitive information about survivors of commercial sexual exploitation, and release could compromise participant privacy and safety. Requests to access the datasets should be directed to Courtney Furlong, cfurlong@hawks.huntingdon.edu.
